# The impact of single-cell genomics on the field of mycobacterial infection

**DOI:** 10.3389/fmicb.2022.989464

**Published:** 2022-09-30

**Authors:** Inês Geraldes, Mónica Fernandes, Alexandra G. Fraga, Nuno S. Osório

**Affiliations:** ^1^Life and Health Sciences Research Institute (ICVS), School of Medicine, University of Minho, Braga, Portugal; ^2^ICVS/3B's—PT Government Associate Laboratory, Braga, Portugal

**Keywords:** single-cell RNA sequencing (scRNAseq), mycobacteria, tuberculosis, *leprae*, single-cell, omics, spatial transcriptomics (ST)

## Abstract

Genome sequencing projects of humans and other organisms reinforced that the complexity of biological systems is largely attributed to the tight regulation of gene expression at the epigenome and RNA levels. As a consequence, plenty of technological developments arose to increase the sequencing resolution to the cell dimension creating the single-cell genomics research field. Single-cell RNA sequencing (scRNA-seq) is leading the advances in this topic and comprises a vast array of different methodologies. scRNA-seq and its variants are more and more used in life science and biomedical research since they provide unbiased transcriptomic sequencing of large populations of individual cells. These methods go beyond the previous “bulk” methodologies and sculpt the biological understanding of cellular heterogeneity and dynamic transcriptomic states of cellular populations in immunology, oncology, and developmental biology fields. Despite the large burden caused by mycobacterial infections, advances in this field obtained *via* single-cell genomics had been comparatively modest. Nonetheless, seminal research publications using single-cell transcriptomics to study host cells infected by mycobacteria have become recently available. Here, we review these works summarizing the most impactful findings and emphasizing the different and recent single-cell methodologies used, potential issues, and problems. In addition, we aim at providing insights into current research gaps and potential future developments related to the use of single-cell genomics to study mycobacterial infection.

## 1. Introduction

The understanding of a cell as the functional unit of life has led to a profound interest in single-cell analysis. This specificity offers unique opportunities to dissect the interplay between intrinsic cellular processes and extrinsic stimuli, and unfold cell-to-cell identity and variability. Besides being referenced as one of the paths for personalized medicine, single-cell studies further allow the understanding of the outcome of an infection, as well as drug resistance as a primary source for the development of clinical tools. Despite the extensive study of the proteome over the years, the transcriptome has thrived in the single-cell field, as it provides insight into changes over a cellular lifetime and in response to external signals (Chambers et al., 2019). From the vast knowledge in this area, single-cell methodologies evolved to included the analysis of DNA, protein, and chromatin modifications (Linnarsson and Teichmann, 2016). Moreover, the latest applications are focused on the simultaneous measurements of two or more modalities, such as genome and transcriptome, transcriptome and methylome, RNA and protein (Linnarsson and Teichmann, 2016), as well as the quantification of these modalities throughout time and space in the cellular environmental niche.

Resorting to *in vivo* reverse transcription (RT) followed by amplification through *in vitro* transcription (IVT), Eberwine et al. (1992) measured the expression of a few individual genes at the single-cell scale. The scaling up of the number of cells and genes analyzed was dependent on further improvement of PCR-based methods (Lambolez et al., 1992) and scale application of micro-arrays or real-time quantitative reverse transcription-polymerase chain reaction (RT-PCR) methods (Peixoto et al., 2004). Taking on a different approach, the bulk RNA sequencing (RNA-seq) method was based on the liaison of millions of cells and the sequencing of this collective admixture, whereby the gene expression profile represents the averaged effect across a large population of cells. However, knowing that gene expression is regulated deferentially across cells and that cells have distinct and specific functions, single-cell sequencing thrived in the field of transcriptomics due to its higher resolution. Building upon PCR and unbiased amplification of cDNA, Tang et al. (2009) performed the first wide coverage single-cell RNA sequencing (scRNA-seq), discovering, and estimatively quantifying many new transcripts that had been overlooked. One year later, Guo et al. (2010) developed a new way to identify cell types in parallel, based on different fluorescence intensity ranges and without cell pre-sorting. Overlooking the field of fluorescent labeling, in 2011, Islam et al. (2011) developed single-cell tagged RT sequencing (STRT-seq), a highly multiplexed method for scRNA-seq, which allowed the obtention of single-cell detail and cell type-specific population averages. The characteristic high throughput and cost reduction were largely dependent on the incorporation of a unique cell barcode and template-switching oligos (TSO), during RT (Islam et al., 2011). To correct amplification bias and improve mRNA amplification, a unique molecular identifier (UMI) was described using a random code for labeling individual mRNA strands and, thereby, distinguishing the original templates from the amplified sequences (Kivioja et al., 2011). Simultaneously, distinct and new techniques combining microfluidics, random capture methods, and *in situ* barcoding arose, allowing a broader implementation, conjugation of steps, and cost reduction.

Following the aforementioned breakthroughs, investments in the technology and improvements in the cost-efficiency have increased the application of scRNA-seq in several fields, including the study of mycobacterial infections. As in other infections, the outcome of an infection by mycobacteria is largely dictated by the interactions between host and microbe. This interaction may cause different outcomes that depend upon the regulation of the host-microbe cell response dynamics and co-evolution (Casadevall and Pirofski, 2000). Based on scRNA-seq advances, a new understanding of both host-pathogen interactions and the development of subsequent treatment strategies has been possible since this technology probes cell-to-cell variability and uncovers host and bacterial responses.

On the whole, this review provides a technical up-to-date summary of the current scRNA-seq techniques and its applications on the study of mycobacteria that are relevant and common human pathogens.

## 2. Technological advances of single-cell genomics

### 2.1. Single-cell RNA sequencing

Each cell from the same organism can have a different phenotype when compared to its neighboring cells. ScRNA-seq enabled the identification of the differences across individual cells in contrast with the previously used bulk RNA sequencing technique (Adil et al., 2021). To achieve the final goal of analyze and extract information from single cells, scRNA-seq has an extensive pipeline ranging from processing the samples to the generation of the gene expression matrix used to perform the data analysis.

Considering all the new doors that scRNA-seq can open, this technique has become widely used; however, new challenges have appeared throughout the pipeline. One of the steps that often generates difficulties is cell isolation and separation. Depending on the final goal of the research, various techniques can be used to make this process more efficient and quick. One of the most used and compatible methods to isolate single cells is flow cytometry with Fluorescence-Activated Cell Sorting (FACS). FACS takes advantage of labeling specific cell surface molecules with a fluorescent tag allowing the isolation and separation of single cells and providing high throughput (Gross et al., 2015). Other technologies, less widely adopted when compared to FACS, are based on custom-built semi-automatic cell collectors (Islam et al., 2011). Although nowadays individual markers can be simultaneously used and cytometers can be interfaced with 96-well and 384-well plates, ensuring the distribution of a single cell per well (Dalerba et al., 2011), the requirement of known specific antibodies and relatively large initial volumes of cell-containing solution (Hu et al., 2016) are relevant disadvantages of FACS. Another disadvantage is the need to have cells in a suspension which cause the loss of tissue architecture and disrupt cellular function and communication (Jahan-Tigh et al., 2012). Additionally, despite the advances made (Baumgaertner et al., 2021), FACS is inefficient for the isolation of cells with largely heterogeneous cell sizes, challenging the identification of rare cell populations (Saliba et al., 2014). Laser capture microdissection (LCM) is an alternative technique which solves some of these problems by allowing precise isolation of individual cells. This method can be classified into two different systems: ultraviolet (UV LCM) or infrared (IR LCM; Hu et al., 2016). LCM does not require cell suspensions, allowing the selection of cells from solid tissue samples and the instigation of heterogeneous tissue sections, which enables the acquisition of information related to cell morphology and structure along with the spatial location (Fink et al., 2006). However, LCM relies on the visual delimitation of cell boundaries, is laborious, and has limited high throughput scalability, making it less attractive to use despite the aforementioned advantages (Gross et al., 2015).

Following the above-mentioned techniques, a novel and less laborious stream of microfluidic or lab-on-a-chip devices arose, being characterized by passive and random cell-capture, and controlled cellular microenvironment features (Young and Beebe, 2010). Microfluidic devices, especially the ones used for single cell separation, can be classified into three main technologies: (1) “trap”-based microfluidics, where cells are retained in small structures present on the microfluidic channel (Carlo et al., 2006); (2) valve-based microfluidics, where cells are isolated by causing deflection on a membrane opening or closing the microfuidic channel (Gómez-Sjöberg et al., 2007); and (3) droplet-based microfluidics, where cells are encapsulated inside well-controlled size aqueous droplets (Brouzes et al., 2009). These microfluidics technologies have different throughput, being the valve-based the one with the lowest throughput and the droplet-based the one with the highest (Edd et al., 2008; Gross et al., 2015).

Currently, the most popular scRNA-seq profiling techniques are built on droplet-based microfluidics. inDrop (Macosko et al., 2015), Drop-seq (Klein et al., 2015), and more recently 10X Genomics Chromium (10X) (Zheng et al., 2017) are the droplet-based microfluidics scRNA-seq methods with the highest throughput (Zhang et al., 2019). These three techniques use similar droplet designs to individualize cells, on-bead primers with barcodes to differentiate individual cells and apply unique molecular identifiers (UMI) to identify molecules and to correct PCR amplification bias. Despite these similarities inDrop, Drop-Seq and 10X techniques take distinct approaches on the microfluidic devices, chosen bead material, and reagent delivery. An example of the disparities among these three technologies is the beads material type. inDrop and 10X systems use beads made of hydrogel, whereas Drop-Seq uses beads made of brittle resin (Zhang et al., 2019). The first two technologies take advantage of the use of a deformable hydrogel, decreasing the probability of encapsulating two cells or two beads in a single droplet. This is possible by conjugating the deformable hydrogel beads with the match between the periodicity of particle flow and drop formation (Abate et al., 2009). It is extremely difficult to achieve 100% of single-bead occupancy due to several factor such as bead size variation. Regardless, both 10X and inDrop protocols are highly effective in capturing single cells (Zhang et al., 2019). Furthermore, the bead material may influence the quantity and density of DNA primers since hydrogel beads allow for large-scale primer immobilization, whereas the brittle resin beads are more limited in this aspect. This will influence the efficiency of mRNA RT and capture, since 10X and inDrop allow the RT to occur within the droplets, confining the reaction to a limited volume enhancing the specificity of cDNA conversion and relative yield (Marcy et al., 2007).

Alternative strategies to the use of emulsion-based technologies are nanowell technologies, which consist in the deposition of cells at random positions, by gravity, inside microwells containing barcoded beads. Nanowell technologies, such as Gene expression cytometry (Cytoseq) (Fan et al., 2015), Seq-well (Gierahn et al., 2017), and microwell-seq (Han et al., 2018), enable massively parallel scRNA-seq, and show advantages over droplet-based microfluidics, including low reagent and sample volumes, and short cell-loading period (Zhou et al., 2021). In 2017, a different study proved the capacity of processing over a million cells using combinatorial indexing. This method is dependent on *in situ* reactions, using multiple rounds of barcoding and mixing, generating combinatorial barcodes and consequent single cell data (Cao et al., 2017; Rosenberg et al., 2018).

Despite the different types of cell capture the overall scRNA-seq follows a similar basic strategy. First, single cells are isolated (cell capture) and lysed. Selecting the right type of cell lysis is important since it may influence the quantity and quality of available RNA (Haque et al., 2017). RT is then performed on RNA to obtain cDNA. Subsequently, the resulting cDNA is amplified by PCR or transcribed by IVT. Finally, the amplified cDNA is used for sequencing library preparation. Each scRNA-seq protocol presents differences in the three main steps: (1) RT; (2) cDNA amplification; and (3) library preparation and sequencing (Saliba et al., 2014).

The RT stage, is characterized by the capture of polyadenylated mRNA species to further perform cDNA synthesis. The polydenylated mRNAs are acquired by using a poly-thymine (poly[T]) sequence primer that will bind to the poly[A] tails present in the mRNA molecules. This step prevent the capture of ribosomal RNA (Haque et al., 2017). An exception to the common use of poly[T] priming is the use of a Designed Primer-based RNA-sequencing (DP-seq) strategy. When compared to the poly[T] priming, DP-seq shows similar transcriptome coverage and technical noise (Bhargava et al., 2013). The main difference between protocols, concerning RT, is the synthesis of the second-strand. This step can be done by poly[A] tailing [used in the protocol described by Tang et al., 2009, CEL-Seq (Hashimshony et al., 2012), and QuartzSeq (Sasagawa et al., 2013)] or template-switching [applied in SmartSeq (Ramsköld et al., 2012) and STRT-Seq (Islam et al., 2011)]. The last one is an alternative strategy that ensures the full transcription of mRNA by covering the premature termination of the 5' transcription in the poly[A] tailing (Saliba et al., 2014). Moreover, during RT some protocols allow the addition of small nucleotide sequences such as UMIs. The implementation of UMIs associated to the cell barcode permits to quantify the number of transcripts in each cell (Kivioja et al., 2011). CEL-Seq (Hashimshony et al., 2012), CytoSeq (Fan et al., 2015), STRT-Seq, MARS-Seq, 10X, Drop-seq, inDrop, and Smart-seq3 (Hagemann-Jensen et al., 2020) are some of the protocols where UMIs can be added (Adil et al., 2021). More recently a technique based on Smart-seq2 was developed, known as FLASH-seq, which combines RT and cDNA pre-amplification (RT-PCR) resulting in a reduction of the time of the protocol (Hahaut et al., 2022). Furthermore, some protocols also implement spike-in RNA controls to facilitate the measurement of accuracy, biases, and sensitivity in RNA-seq procedures (Jiang et al., 2011).

Following RT comes cDNA amplification, where the minute amounts of cDNA are amplified to obtain the required starting material to generate a sequencing library. This can be achieved by either a linear procedure based on IVT, or an exponential procedure based on PCR. The advantage of the PCR is the exponential amplification of cDNAs that allows the amplification of millions-fold in several hours, however the accumulation of primer dimers and other nonspecific products hampers this process, especially during later cycles of PCR. One of the main advantages of IVT is exactly this non-accumulation and the specificity, however the efficacy of cDNA amplification is lower and the process is more time-consuming (Tang et al., 2011). The Tang protocol, SmartSeq, SmartSeq2, and STRT are some protocols that implement PCR. Alternatively, IVT is used in CEL-Seq and MARS-Seq protocols (Hedlund and Deng, 2018). Table 1 has a summary of the protocols used for single-cell preparation.

**Table 1:** Summary table of the most common single-cell sequencing techniques explored in this section, “Single-cell RNA sequencing”.

**Technique**	**Transcript length**	**Preamplification**	**UMI insertion**	**References**
Tang protocol	Full-lenght	PCR	No	Tang et al., 2009
STRT-seq	5' end	PCR	Yes	Islam et al., 2011
Smart-seq/ Smart-seq2	Full-lenght	PCR	No	Ramsköld et al., 2012; Picelli et al., 2013
CEL-seq/ CEL-seq2	3' end	IVT	Yes	Hashimshony et al., 2012
MARS-seq	3' end	IVT	Yes	Jaitin et al., 2014
Drop-seq	3' end	PCR	Yes	Macosko et al., 2015
InDrop	3' end	PCR	Yes	Klein et al., 2015
10x Chromium	3' end	PCR	Yes	Zheng et al., 2017
Smart-seq3	Full-lenght	PCR	Yes	Hagemann-Jensen et al., 2020
FLASH-seq	Full-lenght	RT-PCR	Yes	Hahaut et al., 2022

It divides the information according to the steps of this technology: Technique, Reverse transcription, Preamplification, and UMI insertion.PCR (polymerase chain reaction), IVT (*in vivo* transcription), RT-PCR (reverse transcription-polymerase chain reaction).

The step after cDNA amplification is library construction followed by sequencing using the so called Next Generation Sequencing (NGS) platforms. The library construction is related to the preparation of the RNA strand in a manner compatible with the applied sequencing system. Besides the wide offer of sequencing platforms, such as Complete Genomics (CG) (Lee et al., 2015), Roche (Margulies et al., 2005), Ion Torrent (Rothberg et al., 2011), Pacific Biosciences (PacBio) (Eid et al., 2009), and ABI SOLiD (McKernan et al., 2009), the Illumina platform (Bentley et al., 2008) is the most used, even though the obtained genetic material is compatible with the other platforms (Quail et al., 2012). Illumina provides different technologies ranging from high to low throughput sequencing and is associated with the Nextera library kit preparation (Quail et al., 2012). Despite the advances made to make NGS less expensive, the cost of sequencing is still a barrier to the implementation of scRNA-seq (Grada and Weinbrecht, 2013). This limitation could be minimized by higher-level combinatorial barcoding allowing a more efficient handling of samples (Slatko et al., 2018). Another NGS limitation is the need for an automated routine to employ the use of this technology, specially in the field of clinical microbiology (Besser et al., 2018). Different NGS platforms have specific advantages and disadvantages. The final output of this step, independent of the used technology, is usually a FASTQ file containing the scRNA-seq raw data. This type of file allows to perform the quality control of the sequencing. Several software tools can be used to analyse FASTQ files, being the most popular ones developed for Unix-based operating systems (Chu and Corey, 2012). In general, the analysis pipelines used by those software follow a first stage quality control for the obtained reads, followed by genome alignment and reads quantification. These analysis pipelines then generate a count matrix that will allow additional quality controls and the specific analysis of cell expression profiles (Luecken and Theis, 2019).

**Figure 1:**
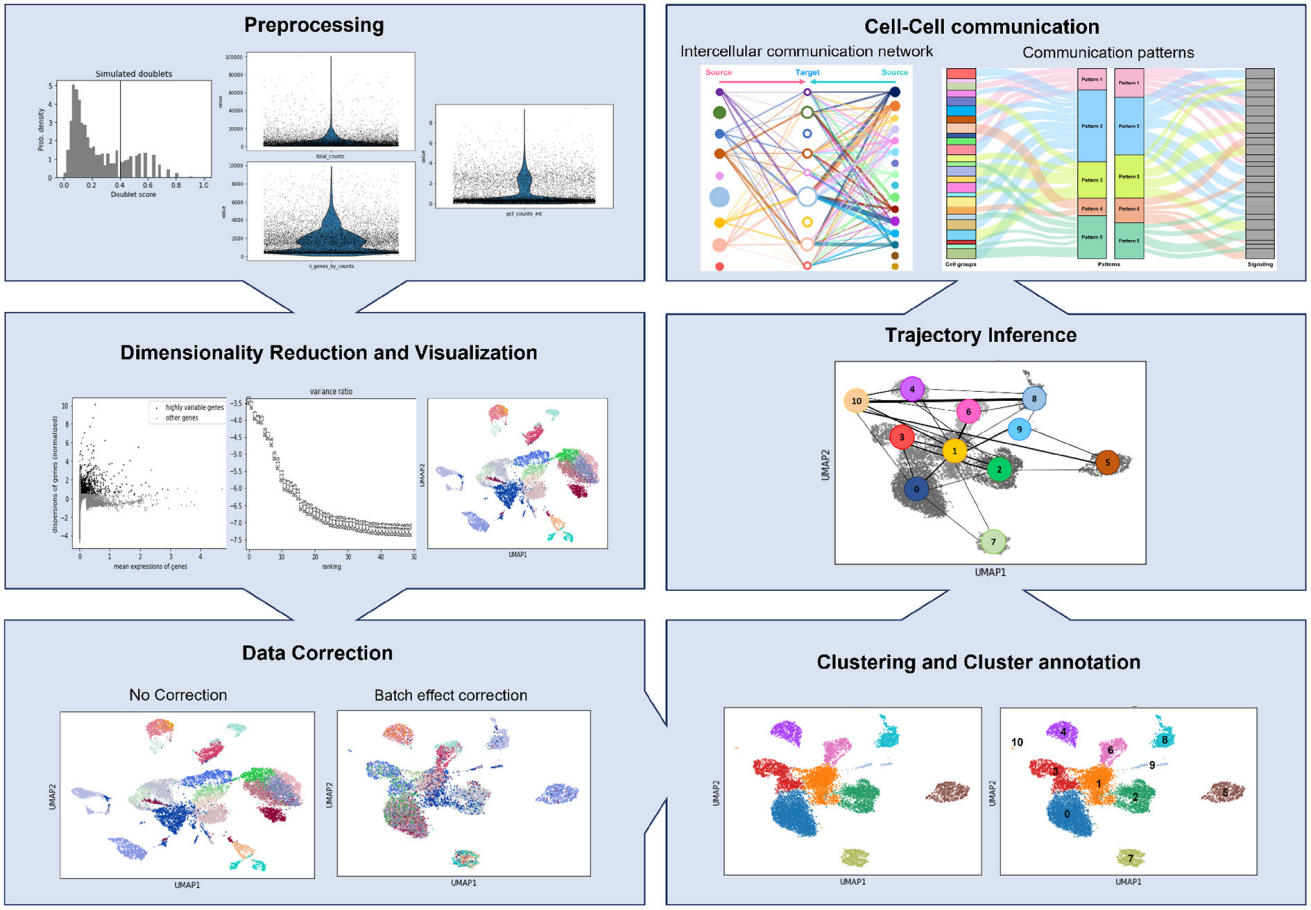
Schematic representation of a scRNA-seq pipeline, showing the outputs of each step stated in this review.

The Python-based tool, Scanpy (Wolf et al., 2018), and the R-based alternative, Seurat (Butler et al., 2018), are the two most used tools for scRNA-seq data analysis. However, the vast availability of different programming languages and pipelines for data analysis causes a lack of standardization (Zappia et al., 2018; Luecken and Theis, 2019). Generally, the analysis pipelines encompass up to eight different steps described below and presented in Figure 1.

### 2.2. Single-cell assay for transposase-accessible chromatin (ATAC) using sequencing

Eukaryotic genomes are hierarchically packaged into chromatin, and the density of this packaging plays a key role in gene regulation. The study of the nucleoprotein structure of chromatin and its packaging is a fundamental research path that has been giving major insights into how chromatin regulation by epigenetic factors controls gene expression and cell programming. This information has come from high-throughput genome-wide methods assessing DNA methylation, transcription factor (TF) occupancy and modification, and chromatin accessibility.

DNA methylation, the first heritable epigenetic mark to be identified, involves the covalent transference of a methyl group to the C-5 position of the cytosine ring of DNA (5 mC). Based on the conversion of single unmethylated cytosine residue to uracil (Bisulfite sequencing technique), single-cell DNA methylation can be analyzed by two similar processes: single-cell bisulfite sequencing (scBS-seq) (Smallwood et al., 2014) and single-cell reduced representation bisulfite sequencing (scRRBS) (Guo et al., 2013). However, due to the non-static characteristics of these processes, it has been shown that 5 mC can be converted to a cytosine (5 hmC). Since the traditional standard techniques for detecting 5 mC cannot discriminate between 5 mC and 5 hmC, and to provide unique insights into the dynamics of DNA methylation turnover and the extent of cellular heterogeneity, in 2016 scAba-seq was presented as a signature to genome-wide detection of 5 hmC marks, using the restrictive endonuclease AbaSI (Mooijman et al., 2016).

The diversity and temporal alteration of cells and tissues in an organism is also dependent on chromatin organization, genome integrity, and gene expression regulation, where protein-DNA interactions play a vital role. In this specific field, Chromatin Immunoprecipitation (ChIP) is a commonly used technique for mapping histone, TFs, and other protein-DNA interactions in the genome. In most ChIP protocols, cells are crosslinked with formaldehyde, then chromatin is fragmented and solubilized, followed by the addition of a highly specific antibody, and finally the purification steps allow the DNA extraction. Subsequently, the bounded DNA is identified by microarray hybridization (ChIP-chip) or deep sequencing (ChIP-seq). Despite the fundamental necessity of crosslinking steps in ChIP, it can promote epitope masking and generate false positive binding sites, when trying to preserve the *in vivo* organization (Kasinathan et al., 2014). Therefore, to tackle these misleading outcomes, ChIP can be performed in low-salt conditions that can functionally fix protein-DNA interactions in a non-covalent manner, since salt competes for electrostatic protein-DNA interactions (Kasinathan et al., 2014).

On the negative side, this technique requires large amounts of input material and yields “averaged” profiles that are insensitive to cellular heterogeneity. Additionally, low levels of non-specific antibody binding pull-down off-target sites and lead to experimental noise. Such scenario justifies the employment of methods based on labeling chromatin from single cells, using droplet-based microfluidics technologies. As previously described, the formation of micron-sized aqueous droplets immersed in a carrier oil acts as ideal microreactors and can be precisely sized to contain an individual cell (Rotem et al., 2015). When first applied, researchers used the method to investigate cell-to-cell variability in different types of regulatory elements by profiling the H3 lysine 4 trimethylation (H3K4me3) and dimethylation (H3K4me2) in a mixed population of mouse embryonic stem (ES) cells, embryonic fibroblasts (MEFs), and hematopoietic progenitors (EML cells) (Rotem et al., 2015). A similar approach, combining droplet microfluidics with single-cell DNA barcoded beads was used to study intra-tumor heterogeneity of chromatin states, based on H3K27me3 histone alteration (Grosselin et al., 2019).

Depending on the quality of the compounds needed, in the first ChIP protocols the use of the purification steps related to centrifugation, generated an enormous loss in the amount of insoluble proteins attached to nuclei sequences (Schmid et al., 2004). Termed as chromatin immunocleavage (ChIC), Schmid et al. reported an alternative strategy to detect binding sites of transcription factors in the genome by targeting micrococal nuclease (MNase) conjugated with protein A (pA-MN), through a specific antibody (IgG and IgG1). The nuclease is activated by the addition of calcium, and fragments of specific binding sites are released into the supernatant for DNA extraction, library preparation, and paired-end sequencing (Schmid et al., 2004). Since IgG and IgG1 ensure the affinity of pA-MN protein, the addition of IgG sepharose beads, to the cleavage reaction, ensures the sequestering of unbound pA-MN or complexes thereof that become unbounded (Schmid et al., 2004).

Based on ChIC, improved techniques such as Cleavage Under Targets and Release Using Nuclease (CUT&RUN) (Skene and Henikoff, 2017) and Cleavage Under Targets and Tagmentation (CUT&Tag) (Kaya-Okur et al., 2019) arose. The CUT&RUN modifications of ChIC were derived by the observation that light MNase treatment released mononucleosomes and TF-DNA complexes, leaving behind oligonucleosomes. A critical modification was based on the immobilization of permeabilized and unfixed nuclei to magnetic beads, allowing the rapid and efficient solution changes, maintaining epitope preservation and accessibility (Skene and Henikoff, 2017). Moreover, the specificity of CUT&RUN is characterized by the performance of digestion at ice-cold temperature and the efficient fractionization based on the solubility of the cleaved fragments (Skene and Henikoff, 2017). Although CUT&RUN can generate high-quality data, it must be followed by DNA end polishing and adapter ligation to prepare sequencing libraries, which increases the time, cost, and effort of the overall procedure. Additionally, the release of MNase-cleaved fragments into the supernatant is not well-suited for application in single-cell platforms (Kaya-Okur et al., 2019). Therefore, CUT&Tag replaces pA-MN for pA-Tn5 fusion protein, pre-loaded with sequencing adapters, that enable chromatin protein binding sites used at PCR (Kaya-Okur et al., 2019). Further alternatives to ChIP including enzyme-tethering methods for unfixed cells, are DamID and ChEC-seq technologies.

Since heterogeneity at the single-cell level extends to chromatin accessibility, transposase-accessible chromatin using sequencing (ATAC-seq) studies this variation, like earlier assays such as DNase-seq, MNase-seq, or FAIRE-seq (Smith and Sheffield, 2020). ATAC-seq allows identifying and sequencing DNA regions that are accessible to external factors as regulatory elements. Promoters, enhancers, and other types of regulatory elements vary spatially, temporally, and among cell types, which influence the binding of transcription factors and the expression of target genes. In scATAC-seq, individual cells are captured and assayed using a programmable microfluidics platform (Fluidigm). The main workflow of this protocol consists in the cut and insertion of sequencing adapters into accessible regions of the genome, through the tag of prokaryotic Tn5 transposase to regulatory regions, following PCR amplification and sequencing by a high-throughput sequencing instrument. Since transposons have been shown to integrate into active regulatory elements *in vivo*, Tn5 is fundamental for the discovery of this locus (Buenrostro et al., 2015). scATAC-seq has been widely adopted due to its efficiency in cost, time, and required amount of necessary samples compared to previous assays (Klemm et al., 2019). However, the epigenetic resolution obtained is only interesting in the context of understanding gene expression. Therefore, RNA sequencing is conjugated with this technique.

### 2.3. Spatial transcriptomics

Even though scRNA-seq is an extremely useful tool to understand single cells' behavior and mechanisms, scRNA-seq by itself does not allow the understanding of how the single cells' genomic and transcriptomic landscapes are spatially organized throughout a tissue (Crosetto et al., 2015). In order to overcome this drawback, a new collection of technologies emerged, known as spatial transcriptomics. These technologies allow the preservation of spatial information since there is no need to dissociate the tissue to obtain single cells for sequencing. This set of techniques is commonly characterized into five groups: (1) microdissected gene expression based technologies, (2) *in situ* hybridization (ISH) based methods, (3) *in situ* sequencing (ISS) based methods, (4) *in situ* capturing technologies, and (5) *in silico* reconstruction of spatial data (Asp et al., 2020).

The main principle of methods based on microdissection gene expression is to isolate and perform RNA extraction of a region of interest, followed by gene expression profiling. The combination of LCM and scRNA-seq gave origin to the first technology of this group, geographical position sequencing (Geo-seq). The use of a laser beam to cut regions of interest within a tissue, selected under a microscope (Emmert-Buck et al., 1996; Simone et al., 1998), allows the scRNA-seq profiling of small regions of the tissue (Chen et al., 2017). Despite being a very robust technique, a disadvantage is the high cost due to the very demanding labor and low throughput (Asp et al., 2020). Another technology is RNA tomography (tomo-seq), which is based on the computational overlap of the RNA information obtained by three cryosections of the tissue with different directions (Junker et al., 2014; Asp et al., 2020). Despite having a good spacial resolution, tomo-seq cannot be used in clinical samples due to the need of having identical tissues (Asp et al., 2020). A very different approach is transcriptome *in vivo* analysis (TIVA), which allows spatial transcriptomics in living cells (Lovatt et al., 2014). This method exposes living tissue sections to photoactivable mRNA capture molecule tags (TIVA tags), that will further enter the cell. This step allows to select only the marked cells by using a photoactivation laser. Moreover, the activated TIVA tags hybridize with the cells' mRNAs, allowing its capture and further analysis. A disadvantage of TIVA is the low throughput and the demand of living tissues (Asp et al., 2020). NICHE-Seq is another method that uses photoactivation, however the tissue recovery is based on the expression of green fluorescent protein (GFP) within an area with a particular microenvironment (Medaglia et al., 2017). This method is dependent on transgenic animals and the GFP detection depends on the intravenous transference of labeled landmark cells. Thereafter, the selected niches can be photoactivated with subsequent dissociation of the tissue and cell sorting followed by scRNA-seq. NICHE-Seq has the advantage of high throughput, however, does not allow to know the exact position of the cell within a niche and cannot be used in human samples since it implies the use of transgenic organism (Asp et al., 2020). Another technique that profiles cells within a niche is ProximID, which allows the understanding of the physical interactions between cells in addition to cellular distance (Boisset et al., 2018). Mild dissociation of the tissue preserve small interacting structures with two to three cells. These structures are manually microdissected making the protocol considerably exhaustive. After that, single cells are subject to scRNA-seq (Asp et al., 2020).

In ISH-based methods, RNA molecules can be visualized straight in their environment by hybridizing labeled probes. One of the oldest ISH-based method, created in 1962, is single-molecule RNA fluorescence *in situ* hybridization (smFISH). smFISH uses short probes to hybridize with different regions of the target RNAs, previously defined. This approach allows quantitative evaluation of the transcripts due to its' greater and more vigorous signaling (Femino et al., 1998). Despite being more efficient than its antecedent method (FISH), smFISH limitation resides in the difficulty of labeling probes with more than five fluorophores. Using large sets of fluorophores makes it difficult to synthesize and purify the RNA (Asp et al., 2020). To overcome this limitation an improved version of smFISH was developed, where a single fluorophore is coupled to ~40 probes (Raj et al., 2008). Despite the enhanced subcellular spatial resolution and sensitivity of this method, in both solely a few number of genes can be targeted due to the spectral overlapping (Asp et al., 2020). In 2014 a multiplex smFISH-based approach that uses sequential hybridization (seqFISH) was developed (Lubeck et al., 2014; Shah et al., 2016b). seqFISH applies various rounds of hybridization, imaging and probe withdraw which allows the identification of singular transcripts. The excessive amount of smFISH probes required in this method makes it really slow and expensive (Asp et al., 2020). To overcome this issue, two new and similar approaches were developed, multiplexed errors-robust FISH (MERFISH) and seqFISH+ (Chen et al., 2015; Eng et al., 2019). The first step in MERFISH is the hybridization of non-readout probes with the target transcript. Thereafter, the fluorescent readout-probes hybridize with the product of the previous hybridization in several circuits of hybridization, imaging, and signal extinction. By changing the usage of smFISH hybridization to just one, and by using multiple readout hybridization, the time of the process can be substantially reduced (Asp et al., 2020). On the other hand seqFISH+, uses primary-probes with flanking regions to target the RNA, followed by a sequence of barcoding cycles with hybridizations (Eng et al., 2019). This approach allows the identification of a higher quantity of unique gene barcodes. Despite being much faster than seqFISH, seqFISH+ still depends on a previous selection of the targets, maintaining the need for a large number of synthesized probes (Asp et al., 2020). A further FISH-based approach is ouroboros smFISH (osmFISH), develop in order to try to bypass optical crowding (Codeluppi et al., 2018). The use of this technique results in a lower number of targets, than the previous mentioned approaches, since this is a non-barcoding method. Here the number of targets is defined by the number of hybridization rounds and imaging is performed after every round, followed by probe elimination and a new hybridization. Besides being quite more laborious than smFISH, the semi-automation of this method overcame this limitation. Additionally osmFISH is more capable of handling larger areas of tissue than the other smFISH approaches, despite its lower multiplex capacity (Asp et al., 2020). A different method was developed to overcome a common inconvenience in FISH-based methods, the high amount of autofluorescence background, that occurs when the tissues are opaque. Single-molecule hybridization chain reaction (smHCR) is an amplified version, where each target has numerous readout probes attached (Shah et al., 2016a). Besides this common limitation, ISH-based methods have spectral overlapping as a disadvantage. This occurs when large groups of different transcripts are detected at the same time. However, the solution to this problem contributed to a computational power limitation caused by the powerful decoding methods applied (Asp et al., 2020). A more recent approach called CosMx was developed by NanoString and uses a spatial molecular imager (SMI) combined with ISH probes and fluorescent readout probes. CoxMx is an high throughput technique that allows to target both RNA and proteins and can be used into fresh-frozen tissue and formalin-fixed, paraffin-embedded (FFPE) tissues. The workflow of this technique is composed by the hybridization of the ISH probe with the target, followed by several rounds of readout probes, containing 16 sets of reporters (He et al., 2021). Leaving behind the FISH-based approaches, RNAscope is based on designing probes. Here, target RNA transcript binds to two adjacent Z-probes in order to construct the necessary binding site to allow further hybridization of the molecule responsible for amplification (Wang et al., 2012; Corporation, 2019). A limitation of RNAscope is the low multiplex level (Asp et al., 2020). DNA microscopy is a completely different approach, presented as an optic-free nucleotide mapping technique (Weinstein et al., 2019). This method relies on thermodynamic entropy instead of physical capturing, however until 2020 this has only been demonstrated on cultured cells with a small-scale subgroup of transcripts (Asp et al., 2020).

Another *in situ* approach is the ISS-based technologies, where RNA is sequenced straight from the cell within the tissue of interest. Here the location of the transcript is achieved with subcellular resolution, but to reach sufficient signal for imaging, micrometer- or nanometer-sized DNA balls are used to amplify the signal, which is jeopardized by the inherent spatial limits of the cell (Asp et al., 2020). The first ISS-based method was ISS using padlock probes, which are single strand molecules of DNA complementary to the target cDNA (Ke et al., 2013). In this technique mRNA is RT into cDNA to allow the binding of the padlock probes within the tissue section of interest. This method has two different approaches, one uses padlock probes that bind to the cDNA leaving some gaps amidst the ends, and another where, the ends of the molecule are adjacent to each other. In both methods the ends of the DNA molecule bind to form a circle of DNA, however in the gap version, an intermediate step is needed to fill the gap. Thereafter, cDNA amplification is performed by rolling-circle amplification (RCA), giving rise to RCA products (RCP) which will be decoded by using sequencing-by-ligation (SBL) method. The difference between gap and non-gap approaches cause different outcomes, with the gap approach directly allowing the reading of RNA, while the non-gap approach has a higher sensitivity (Asp et al., 2020). A new approach based on the ISS padlock probes gap method was develop few years after to overcome the limitation of the loss of sensitivity and allowing a higher number of base pairs (bp) in the gap, known as barcode *in situ* targeted sequencing (BaristaSeq) (Chen et al., 2018). Despite the improvement on the RCP quantification BaristaSeq uses sequencing-by-signaling (SBS) instead of SBL, which has a higher signal-to-noise fraction. More recently, a ISS barcoded approach was develop in order to overcome noise caused by sequencing and the cDNA conversion efficiency barrier limitations, from previous methods. Spatially resolved transcript amplicon readout mapping (STARmap) combines the use of padlock probes with a second primer to target the site next to the probes, allowing to bypass the RT step and overcome the previously mentioned limitations (Wang et al., 2018). Then amplification is performed by RCA, forming nanoballs, which are single-stranded DNA nanometer-sized products, instead of micrometer-sized RCPs. Thereafter, the nanoballs are embedded in a hydrogel-tissue, followed by a modified SBL. This approach enables to visualize the location of the cells in 3D; however, is limited to a narrow amount of targets, and can only be used in tissue sections with a thickness comprised between 100 and 150 μ (Asp et al., 2020). To overcome the requirement of previous knowledge of the targets and its' selection, in which all the previous methods are based on, a new approach was develop. Fluorescent *in situ* RNA sequencing (FISSEQ) is a non-target needed method that allows to capture every RNA species (Lee et al., 2014). In FISSEQ a mixture of modified and typical amine-bases combined with tagged RT primers is used, to perform the cDNA synthesis that after RCA will give rise to nanoballs, followed by SBL. The position of the nanoballs is known due to the cross-linking with the cellular environment. Despite overcoming previous limitations, in this method only a random fraction of the nanoballs is sequenced (Asp et al., 2020).

A different approach for spatial transcriptomics is *in situ* capturing technologies based on *in situ* capture of the transcripts and *ex situ* sequencing and allow the analysis of the transcriptome in an unbiased way (Asp et al., 2020). The first method developed using this approach was spatial transcriptomics (ST), where RT is executed *in situ*, followed by cDNA-mRNA sequencing *ex situ* (Ståhl et al., 2016). In ST the spatial information is achieved by placing a tissue section onto glass slide with oligo(dT) barcoded RT primers containing the coordinates. ST limitation is its resolution, however, in 2018 10X Genomics acquired ST and improved the protocol giving rise to 10X Visium (10x Genomics, 2019; Asp et al., 2020). A similar approach is Slide-seq, but instead of having the RT primers printed in the glass, they are dispensed on the top of the glass slide (Rodriques et al., 2019). In Slide-seq, SBL is performed *in situ* since the position of the barcoded beads are unknown due to their random distribution. Slide-seq has a sensitivity limitation that demands the usage of scRNA-seq data to accurately map the cell types (Asp et al., 2020). Although Slide-seq has a higher resolution than ST a new method with even higher resolution was lately developed. High-definition spatial transcriptomics (HDST) uses tinier beads than the previous methods and barcoded beads with RT primers randomly distributed into the bead array (Vickovic et al., 2019). Despite its higher resolution, HDST also needs the SBL to be performed *in situ* and requires scRNA-seq data to help the mapping process (Asp et al., 2020). A completely different approach is APEX-seq, based on profiling RNA subcellular regions inside living single-cells (Shah et al., 2016b; Fazal et al., 2019). However this method uses cell lines expressing the APEX2 enzyme on the subcellular area of interest, which makes it ineffective to be used in regular tissue samples (Asp et al., 2020). A different and more complete approach is GeoMx, which has the ability to spatially profile proteins in addition to mRNA, allowing sub-cellular and single cell analysis, however in different tissue sections (NanoString, 2019). In this technique it is needed to manually select different regions of interest (ROI) that will further be subjected UV light in order to release the target probes associated with the barcoded tags, for both RNA and protein experiments. GeoMx has limitations such as low multiplexing capacity, regional analysis of the tissue in an unbiased way and for small ROI (≈ 10μm) it has a low efficiency in protein detection (Van and Blank, 2019; Asp et al., 2020). With the same goal, but using a different approach, microfluidic deterministic barcoding in tissue (DBiT-seq) was developed (Liu et al., 2020). In this method, a microfluidic chip containing channels with barcoded tags for both mRNA (oligo-dT tags) and protein capture (antibody tags) is planted on top of the tissue region of interest (Asp et al., 2020).

Finally, it is also possible to perform spatial transcriptomics *in silico* which uses computational methods to assign a spatial location to each single cell from a dataset based on their gene expression. *In silico* transcriptomics algorithms can be classified in two groups, the ones that use reference maps and the ones that rely on assumptions based on gene expression traits without using a reference map (Asp et al., 2020). The first algorithms developed use computationally generated ISH reference maps of smaller regions within the tissue of interest, and a spatial location is attributed to each cell using a small group of descriptive genes (Achim et al., 2015; Satija et al., 2015). Despite being reliable approaches to be used in well-described and defined tissue structures and organisms, it is quite difficult to apply these methods in more complicated structures. Furthermore these approaches cannot be used in clinical samples and is limited to already existent ISH maps (Asp et al., 2020). Methods that do not use a reference map allow to overcome the previous stated main limitation. NovoSpaRc enables *de novo* spatial positioning without any reference map (Nitzan et al., 2019), by taking in account assumptions of how gene expression changes in the tissue and that proximal cells have a more identical transcriptional profile. Although being a promising method, further studies need to be done (Asp et al., 2020).

Despite being a really helpful tool to understand how single cells' landscapes are spatially organized throughout a tissue, spatial transcriptomics by itself cannot achieve a deep single cell transcriptomics resolution. To overcome this sort off limitation, it is possible to combine scRNA-seq data, from public repositories, to help understand the distribution and architecture of cells. To integrate scRNA-seq data and spatial data, there are two distinct methods: mapping and deconvolution. Mapping can be divided into two branches: (1) mapping each cell to a precise region of the tissue and (2) mapping cells based in scRNA-seq cell types and subtypes. Deconvolution is used to untangle subpopulations of cells mixed with transcripts of mRNA (Longo et al., 2021).

### 2.4. Temporal transcriptomics

Temporal transcriptomics can be incorporated in the scRNA-seq and spatial transcriptomics analysis. This type of analysis predicts cells' trajectory and cells' lineage, allowing to see the progression of cells fate during time. Although the efforts made the challenge to introduce temporal information on scRNA-seq data prevails due to the need of cell lysis to perform sequencing, which makes it impossible to sequence the same cell again in a different progression stage. This technique is divided into two branches: (1) differentiation trajectory and (2) lineage tracing.

Differentiation trajectory reconstruction is used when analysing time scales ranging from hours to days. This one diverges into three smaller branches which are the usage of intronic reads to predict changes in expression over time, pseudo-temporal cells' arrangement, and RNA metabolic labeling (Olivares-Chauvet and Junker, 2020). The intronic reads uses a vector that can predict the next stage of a cell based on their expression (La Manno et al., 2018). This method, also called RNA velocity, has the enormous advantage of no additional steps needed during the scRNA-seq experiment. However, limitations include the absence of quality control, which disable a more specific transcriptional dynamic analysis, and the fact this method can be solely applied to a portion of the identified genes (Olivares-Chauvet and Junker, 2020). Pseudo-temporal cell ordering is possible to use when a considerable number of cells are captured during sequencing. Here cell trajectory is predicted by connecting neighboring cells following the dimensionality reduction or by using k-nearest neighbors graph (Trapnell et al., 2014; Setty et al., 2016). Furthermore this method allows the characterization of differentiation events in early developmental stages if the sequenced cells are taken at different time points (Wagner et al., 2018). However, this method does not allow the visualization of the cellular trajectory differentiation direction in contrast with the previous method. Contrarily to the intronic reads based method, RNA metabolic labeling enables the analysis of specific transcriptional dynamics such as transcriptional, splicing, and decay rates. Despite all the advances this method limitation is the necessity to incorporate a new step into the scRNA-seq pipeline to add the labeling (Olivares-Chauvet and Junker, 2020).

When there is a need to perform temporal transcriptomics using larger timescales, it is preferable to use lineage tracing. This method was first performed manually by following the development and cell division of a given organism under a microscope. With the same principle, this method allows to perform lineage tracing from extremely specific cellular niches to all the existent cells in an organism by using high-throughput strategies (Olivares-Chauvet and Junker, 2020). The combination of cellular changes on DNA and cell type identification are used as lineage markers (Ju et al., 2017; Wangsanuwat et al., 2019; Olivares-Chauvet and Junker, 2020). Besides the differences among the high-throughput methodologies used in lineage tracing, the majority of the most recent methodologies take advantage of the CRISPR/Cas9 system to break the double-strand into the transgene. Thereafter, the repair mechanisms may lead to indels near the cut spot, that can be used as barcodes (Olivares-Chauvet and Junker, 2020).

## 3. The burden of mycobacterial diseases

Stated by the World Health Organization (WHO), Tuberculosis (TB), Leprosy and Buruli ulcer (BU) are the three most common mycobacteria causing disease in humans. Looking at the WHO-published numbers, there were 127,558 new leprosy cases detected globally in 2020 (World Health Organization, 2022b), whereas in the same year, 10 million TB (World Health Organization, 2021), and 1,258 BU cases were reported (World Health Organization, 2022a). Incidence and prevalence of these diseases differs considerably per country, noting that developing countries bear the biggest brunt of both new cases and that of patients undergoing treatment. Despite an apparent reduction in the number of cases of these diseases in 2020, it is important to take into account the disruption of the WHO programs and control efforts, caused by the COVID-19 pandemic. These diseases continue to be major global health concerns, due to a marked increase in the number of susceptible individuals (Bruchfeld et al., 2015; Chao et al., 2020; Amoako et al., 2021) and associated prejudicial direct and secondary effects on global health and economy (Saraya et al., 2012). Moreover, the increasing antimicrobial resistance limits the efficacy of the reduced number of compounds available for treatment, stressing the need for expansion of surveillance and development of novel therapeutic and disease control strategies (Owusu et al., 2016; Gygli et al., 2017; Cambau et al., 2018).

Since the beginning, humans have been exposed and coexisting with bacteria from the genus *Mycobacterium*, mainly through contact with soil and untreated water. These bacteria mainly infect humans *via* the respiratory and gastrointestinal tract or skin. Although commonly known as a generalist environmental mycobacteria with capacity to cause disease in certain situations, a minority of mycobacterial species has gone through a long evolutionary process, evolving to a slow-growing pathogenic lifestyle (Gutierrez and Somoskovi, 2014), with *Mycobacterium tuberculosis* and *Mycobacterium leprae* standing out. *M. tuberculosis* is responsible for TB, which is a primarily pulmonary disease initiated by the deposition of the *M. tuberculosis, via* an airborne route, into lung alveolar surfaces (Smith, 2003). Macrophages are the first cellular line of protection, due to the expression of a large array of pattern recognition receptors, being present in pulmonary tuberculous granulomas. Nonetheless, these myeloid cells, specifically alveolar macrophages (AMs), present a dual role in TB also contributing for the spread of *M. tuberculosis* during the course of disease. On the other hand, dendritic cells act on antigen presentation in lymph nodes, in which a T-cell response can subsequently be developed (Silva Miranda et al., 2012). The delay between the arrival of T cells at the site and the establishment of infection may contribute to the inability of the host defenses (Silva Miranda et al., 2012). All these signaling events lead to the formation of a granuloma, the hallmark of tuberculosis (Mayer-Barber and Barber, 2015). TB can present itself as a dynamic panoply of conditions, ranging from an asymptomatic infection to a life-threatening disease (Pai et al., 2016). In addition to host-related factors bacterial diversity might also have a relevant role in orchestrating immune responses to direct the distinct TB severities (Bastos et al., 2016; Yruela et al., 2016; Sousa et al., 2020). There are several treatment regimens recommended for TB ranging in duration from 4 to 9 months and relying on combination chemotherapy with drugs that include isoniazid, rifampicin, Ethambutol, Pyrazinamide, and others. Unfortunately, the appearance of monoresistant-TB, multidrug-resistant tuberculosis, extensively drug-resistant tuberculosis, and total drug-resistant tuberculosis, is increasing to a point of critical obstruction of the efficacy of currently available drug regimens (Hameed et al., 2018). This phenomenon is fueled by factors such as the long treatment duration, improper use of antibiotics, evolution, and transmission of drug-resistant *M. tuberculosis*, limited access to drugs or diagnostic tools making imperative the development of new TB preventive and therapeutic strategies (Bastos et al., 2018; Rocha et al., 2021; Santos-Pereira et al., 2021).

On the other hand, *M. leprae* is the causative agent of Hansen's disease, also widely referred to as leprosy. As classified by the Ridley-Jopling System, the disease spectrum of clinical and histopathological forms varies between two poles, namely tuberculoid (TT) or lepromatous (LL), depending on the immunological response of the host (Walker and Lockwood, 2007). TT presents not only a *M. leprae*-specific Th1 response, but also a T_*H*_17 response that limits *M. leprae* multiplication; in contrast, LL is characterized by a Th2 and T regulatory responses that allows bacterial dissemination instead of control (Fonseca et al., 2017). This mycobacterium causes a chronic infection in humans, mainly affecting the peripheral nerves and skin. In addition, it can further affect sites such as the eyes, mucous membranes and bones, producing a spectrum of clinical phenotypes (Walker and Lockwood, 2007; Graham et al., 2010; Polycarpou et al., 2013). In the skin, *M. leprae* has an affinity for keratinocytes, macrophages, and histiocytes, while in peripheral nerves, *M. leprae* can target Schwann cells giving rise to axonal dysfunction and demyelination, which leads to sensory loss and disabling or deforming conditions (Polycarpou et al., 2013). The treatment of Hansen's disease involves a combination of antibiotics, with dapsone and rifampicin typically being conjugated for 6 months. Furthermore, lepromatous forms of the disease imply the addition of clofazimine to the treatment (Belachew and Naafs, 2019). Since *M. leprae* does not grow *in vitro* the resistance to antibiotics in laboratory has not yet been widely assessed. Notwithstanding, it has recently been disclosed an emergence of resistance to rifampicin (Cambau et al., 2018).

Although the mycobacteria responsible for tuberculosis and leprosy are recognized as obligate pathogens of humans, environmental mycobacteria such as the ones responsible for BU disease and non-tuberculous mycobacterial diseases are gaining recognition and relevance. BU is an indolent necrotizing and slowly progressive disease caused by *Mycobacterium ulcerans*, whose key pathogenic factor is the lipid toxin mycolactone (Kumar et al., 2015). During the progression of *M. ulcerans* infection, mycolactone is responsible for tissue necrosis and inhibition of the host' immunity. Since 2004, the WHO issued treatment guidelines recommending the combination of the antibiotics rifampicin and streptomycin for 8 weeks associated with other supportive treatments, namely wound management and physiotherapy (Kumar et al., 2015; Converse et al., 2018). In 2017, the WHO Technical Advisory Group on BU replaced streptomycin by oral clarithromycin, considering that injections of the former were painful and could cause ototoxicity (Yotsu et al., 2018). In the three mentioned diseases vaccination with *Mycobacterium bovis* bacillus Calmette-Guérin (BCG) is a safe, but only partially effective, prevention measure (Phillips et al., 2015; Coppola et al., 2018).

To clarify the pathogenesis of these diseases, it is vital to characterize the host-pathogen interactions in the course of the infection. Although traditional phenotypic measurements and bulk transcript analysis can provide insights into pathogenesis, the heterogeneity of individual cell populations and its contribution to disease progression is challenging to be analyzed (Huang et al., 2021). However, the emergence of scRNA-seq with high resolution and scale now allows transcriptome analysis of thousands of individual cells. Thus, the use of scRNA-seq has refined the knowledge of the human cell landscape and has driven progress in various areas including immunology, developmental biology, oncology, and infectious diseases (Huang et al., 2021). In the latter, single cell approaches are imperative to obtain insights on the evolution of infections, by identifying susceptible cell types, examining infection dynamics, studying immunological changes, discovering biomarkers, and, ultimately, contributing to unravel novel treatment strategies (Luo et al., 2020).

## 4. Application of single-cell genomics to study mycobacterial infection

A bacterial infection is characterized by host-microbe interactions, that may have different outcomes depending on the regulation of the host-microbe relationship and its' coevolution. Looking at its' global impact, infection caused by mycobacteria present a substantial threat to public health and specifically in patients with defects in cell-mediated immunity (Brown, 2008). The continuous spread, profound negative effects, and inexistence of an efficient cure are related to the diverse number of strategies employed by these mycobacteria to infect, escape the immune system, proliferate, and establish in host cells. Based on scRNA-seq advances, a new understanding of both host-pathogen interactions and the development of subsequent treatment strategies has been made possible, since this method probes host's cell-to-cell variability and uncovers in a very sensitive way the host's immune response to a bacterial threat. Throughout this section the main findings using scRNA-seq on the two most relevant intracellular mycobacterial infections, responsible for tuberculosis or leprosy, will be explored.

When facing the progression of an infection, the activity and response of heterogeneous immune cells play a central role in important biological processes, such as pathogen identification, antigen presentation, immune response, threat neutralization and elimination, and tissue recovery. Since scRNA-seq can define the transcriptomic heterogeneity of a complex community of cells during the infection process, obtain high-throughput data of cellular phenotypes, and assign robust identity classifications to cell populations, this approach deepens the understanding of infectious disease mechanisms. To initially illustrate these strengths in the mycobacteria field and studying, specifically, the *M. tuberculosis* infection, Gierahn et al. (2017) developed and used seq-Well to profile thousands of primary human macrophages exposed to tuberculosis. Although five clusters were initially defined, lower transcript capture and high mitochondrial gene expression lead the authors to focus on three distinct clusters in their macrophage culture that passed quality control. As expected from previous literature, infection of macrophages with *M. tuberculosis* resulted in pronounced shifts in gene expression, such as genes related to Toll-Like Receptors (TLRs), in a response that was shared by all the discovered clusters. Interestingly, the authors also reported cluster-specific responses to the exposure to *M. tuberculosis*. Differential expression of cell growth genes was only found in cluster 1, metabolism-associated genes in cluster 2 and genes involved in hypoxia in cluster 3 (Gierahn et al., 2017). Overall, this study suggests either distinct origins or adaption to different microenvironments by the defined clusters, uncovering a level of cellular heterogeneity in response to *M. tuberculosis* infection that was previously overlooked (Luo et al., 2020).

In a more recent study, Esaulova et al. (2021) advanced an in-depth analysis of the immune landscape in the lungs of healthy, latent TB infection (LTBI), and pulmonary tuberculosis (PTB) in macaques, using a droplet-based microfluidics single-cell technology. Specifically, major differences in lung T cell activation state were observed. T cells from control and LTBI macaques were predominantly represented by naive CD4^+^CD8^+^ T cells and effector memory CD4^+^/CD8^+^ T cells, whereas PTB lungs were enriched in activated CD4^+^/CD8^+^ T cells. Although the common expression of IL7R, TCF7, and LEF1 genes and the low expression of activation markers, a new subset of naive T cells was identified in PTB patients. This identification depended on the single-cell differential expression analysis of heat shock proteins, including HSP1A and DNAJB1. Regarding the myeloid cell family, three differential clusters of macrophages were sorted by this technology. In the lungs of control and LTBI macaques, “CD163^+^MRC1^+^ macrophages” were distinguished by classic markers of alveolar macrophages (AM)-like population identifiers, namely MARCO, MERTK, and APOE. The second cluster formed, which also expressed CD163^+^MRC1^+^ and presented a higher expression of TREM2, C1Q, and TREM176A/B genes, was the “CD163^+^MRC1^+^TREM2^+^ macrophage” subgroup. This subgroup was found in the lungs of healthy macaques. Finally, “IFN-responsive macrophages” was CD163^+^MRC1^low^ and uniquely located in the lungs of macaques with PTB. These macrophages produce proinflammatory proteins and express suppressive molecules such as IDO1. Importantly, lung CD27^+^ Natural killer (NK) cells were associated with protection during latent TB. Contrarily, the influx of plasmacytoid dendritic cells (pDCs), Interferon (IFN)-responsive macrophages, and activated T cells into the lung were the three defining features associated with active pulmonary TB (Esaulova et al., 2021). This study demonstrated the capability of scRNA-seq to currently distinguish different and novel populations of cells based on the transcription readings obtained per cell and therefore establish significant differences between control and stages of the disease.

Although capable of distinguishing between different stages of the disease, single-cell analysis can be useful to study and evaluate the cellular and molecular footprint particularly related to disease progression. Nathan et al. (2021) focused on the single-cell sequencing of only memory T cells isolated from PBMC of TB progressing cases. Although, 31 memory T cell states were sorted (four major clusters—CD4^+^, CD8^+^, CD8^+^CD8^+^CD4^-^CD8^-^), the differential abundance and function of T_*H*_17 subset between progressors and non-progressors donors directed the study to its' deep analysis. Although dependent on age, sex, winter blood draw, and proportion of European genetic ancestry, this disparity may either be a long-term consequence of prior TB disease or predisposition to TB disease progression. The T_*H*_17 subset is marked by a CD4^+^CD45RO^+^CD26^+^CD161^+^CCR6^+^ which confers cytotoxic and proinflamatory properties (Nathan et al., 2021). Another biomarker of the progression of this disease was proposed by Bossel Ben-Moshe et al. (2019). Based on the knowledge gained from the single-cell analysis of infected PBMCs, the authors trained a dynamic deconvolution algorithm to achieve the same resolution in bulk measurements as in single-cell sequencing. It was observed that TB patients, when compared to LTBI and controls, expressed less NKT cell activation and higher monocyte activation. Furthermore, the different signature of infected monocytes between progressors and non-progressors individuals, suggests the capability to identifying LTBI individuals with higher risk to develop active TB (Bossel Ben-Moshe et al., 2019).

Another key aspect to understand host-pathogen interaction consists in uncovering the distinct regulation of the overall immune response in physiological vs. pathological conditions. Cai et al. (2020) focused on this particular interaction, by comparing PBMCs isolated of LTBI and TB vs. healthy control (HC). Using a droplet-based microfluidics single-cell technology, three major cell types (T cells, B cells, and myeloid cells) were prominent, with 29 subsequent subsets being found. Given that the cellular composition of PBMCs changes during pathological stress, an abundance of CD14^+^ myeloid cells had been expected and was witnessed, with three subgroups of CD14^+^ pro inflammatory cells being specifically enriched in TB clinical cases, when compared to HC and LTBI. Sub-cluster analysis also revealed two distinct clusters of circulating NK cells, T2 (FCER1G, GZMB, GNLY, SPON2, PRF1,CD7, MYOM2, and KLRF) and T7 (MH, FGFBP2, GNLY, and selectively expressed KLRC). These clusters allowed the understanding of differential progression of the disease, since T2 gradually decreased from LTBI to TB, compared to HC; while T7 was present in a higher frequency in LTBI. Importantly the depletion of CD3^-^CD7^+^GZMB^+^ NK cells in TB, when compared to HC and LTBI, was recognized as a distinct marker of the different stages of tuberculosis. The authors further analyzed the frequency of this subset after anti-TB treatment, noticing an increase of CD3^-^CD7^+^GZMB^+^ after 3 months, corroborating the results found when using single-cell and flow cytometry (Cai et al., 2020). Overall this article suggests single-cell as a tool to characterize the flow of the disease by analyzing NK cell alteration in blood samples.

To complete the profiling of the overall immune cell response between physiological and infection conditions it is crucial to specifically investigate the differences in cell susceptibility during infection. Looking into the lungs of tuberculosis infected mice, Pisu et al. (2021) developed a multimodal scRNA-seq protocol. To fully assess cell susceptibility during infection, single-cell transcriptional data was used to determine the direct correlation between fluorescence (hspx'::GFP) and environmental stress, sensed by the bacteria. Although previous work carried out by the authors had shown that, at a population level, alveolar macrophages (AMs) were more permissive to bacterial growth than the interstitial macrophages (IMs), the in depth single-cell data obtained showed that both IM and AM lineages each contain discernable and sensitive distinct subpopulations. The authors highlighted only three populations of IM (IM_1, IM_2, and IM_3) due to the low expression of IM_4. The additional pseudotime analysis suggests that the latter subset represent a transitional phenotype between the IM_3 and IM_1. The IM_1 subpopulation is characterized by overexpression of cyclooxygenase2 and Il1β, both of which are known to be involved in a pathway that promotes a protective response in *M. tuberculosis*-infected mice and up-regulates genes associated with resolution of inflammation. On the other hand, IM_2 represents a more heterogeneous cluster, exhibiting a Nrf2 signature that has been associated with a reduced inflammatory response, sensed by the hosted bacteria. Finally, the IM_3 subpopulation is relatively homogeneous and up-regulates the expression of the Nos2 protein and pro-inflammatory genes. Concerning the AM lineage, different subpopulations were characterized by different expression of proinflammatory responses. The AM_1 population included a subset, designated AM_Pro-infl, that expressed high levels of Nos2, and showed up-regulation of proinflammatory mediators. Both AM_1 and AM_3 populations shared a common signature, usually associated with M2 polarization and up-regulation of fatty acid metabolism. These cells appear to be permissive to *M. tuberculosis* growth as detected by the low expression of the hspx′:: GFP reporter. A similar population of inflammatory AMs was also present in the AM_2 cluster, being closely related to IM, with respect to a transcriptional profile upon infection with *M. tuberculosis*. Finally, the transcriptome of the AM_4 cells revealed a phenotype of cell division, suggesting that this subset is responsible for the maintenance and replenishment of the AM population (Pisu et al., 2021).

In addition to the capacity to distinguish different and rare cell populations, while simultaneously correlating this information with the expressed gene profile, the authors also applied scATAC-seq to assess changes in chromatin organization of AMs and IMs. The data analysis showed that chromatin-accessible patterns in IMs and AMs of BCG-infected mice presented similar immunological responses to those observed *M. tuberculosis*-infected mice. The authors then studied the surface markers of infected cells, by assessing the level of correlation between single-cell and flow-cytometry data. The results confirmed that CD11c^low^ AM and CD11c^low^ IM populations presented an increase in the median fluorescence intensity of the GFP signal. Based on this evidence the authors suggested that CD11^low^ cell populations might harbor *M. tuberculosis* bacilli, which can be correlated to a higher degree of drug tolerance, being this a finding with potential implications for TB treatment (Pisu et al., 2021).

In regards to leprosy, scRNA-seq data from four leprosy lesions has been published by Hughes et al. (2020), focusing on the development and application of a second-strand synthesis-based scRNA-seq technique called Seq-Well S3. In this study, the authors observed enhanced enrichment of TCR sequences; a T-cell subset enriched for ROR-γT expression (suggesting the primary role of Th-17 cells in bacterial control); a unique population of macrophages defined by expression of extracellular proteases; as well as elevated expression of IFN-γ. The latter molecule controls transcription programs in Langerhans cells, which, by comparison with normal skin biopsies, showed elevated expression of pro-inflammatory molecules (IDO1, STAT1, HCAR3, and MHC class I). Finally, different groups of fibroblasts were classified based on the expression of POSTN and MMP11 molecules and high amounts of SFRP2, PRSS23, and IL6 (Hughes et al., 2020). On other hand, Mi et al. (2022) focused on analysing two main scenarios: (1) full comparison between data from leprosy patients and healthy controls and (2) the analysis of the intercellular crosstalk between immune cells. The authors presented a detailed host-immune landscape against *M. leprae* in Lepromatous leprosy (L-lep), through the application of droplet-based microfluidics single-cell technology on skin lesions and PBMCs. As a result, part of the molecular mechanism by which *M. leprae* escapes the immune response to reside and proliferate within host cells was clarified. Nine skin immune cells subtypes were revealed, including B cells, CD4^+^ T, CD8+ T, NK, mast cells, Langerhans cells, Mac_LIPA, Mac_FCN1, and CD1C^+^ DC. In respect to L-lep lesions, CD8^+^T cells revealed a significant upregulation of TIGIT and LAG3, implying the exhaustion of this type of cells. This finding was corroborated studying the intercellular communication, where the enhancements of interactions between CD8^+^T cells and antigen-presenting cells (CTLA4/CD86, PDCD1/PDCD1LG2, and TIGIT/NECTIN2) were noticeable. Regarding PBMCs from L-lep patients, alterations were observed by the expansion of the Treg cell subset. In addition, there was an increase of communications between Tregs and another cell type [mediated by CTLA4/CD86, PDCD1 (PD-1)/PDCD1LG2, LGALS9/HAVCR2 (TIM-3), PVR/TIGIT, TIGIT/NECTIN2, and TIGIT/ NECTIN3], in a way that Treg could exert their characteristic immunosuppressive function. Finally, the upregulation of APOE led the authors to suggest the involvement of this molecule in the pathogenesis of the infection (Mi et al., 2022). All things considered, the knowledge of the immune signatures and immune cell deficiencies associated with Leprosy was depended by single-cell analysis.

Knowledge on the granuloma architecture and its molecular spatial distribution is fundamental for the better understanding of correlates of immune protection against *M. tuberculosis*. The function of the granuloma is to sequester and degrade microbial pathogens that have evaded the early immune response. Gideon et al. (2022) applied Seq-Well platform for the study of 26 cynomolgus macaques' granulomas, distinguishing them between low and high Colony-forming unit (CFU) counting. After performing all the bioinformatic data care steps, high-quality transcripts were used for downstream analysis. This led to the identification of 13 general immune cell type clusters across all granulomas, further detecting subclusterings among the T/NK and macrophage cluster. Due to the parallel study of the bacterial burden it was possible to observe that an higher bacterial burden present higher proportions of plasma cells and mast cells. The molecular and cellular interaction in this context reflect fibrosis, metabolic remodeling, and angiogenesis associated with immune attempts at wound healing. On the other hand, T/NK cells were more abundant in low-burden granuloma, being identified clusters of cells with transcriptional features of both type 1 and 17 T cells associated with a coordinated immunity (Gideon et al., 2022). As a drawback, this study makes it impossible to investigate the structural organization of the granuloma. Furthermore, single-cell technology requires high quality cells and given the heterogeneous environment within the granuloma, as well as the presence of necrotic debris in the caseous center, the cell sample may be unrepresentative or even biased for highly expressed populations. Therefore, by integrating scRNA-seq with spatial sequencing, it was possible to delineate the cellular and molecular structure of the organized granuloma in leprosy. Ma et al. (2021) focused the study on T-lep and reversal reactions (RRs), which is a dynamic process characterized by the transition from a L-lep scenario toward self-limiting tuberculoid leprosy (T-lep). A mixture of M1-like and transitional macrophages was observed at the core of the organized granuloma in RR and T-lep lesions. The pseudotime trajectory analysis of the leprosy scRNA-seq data suggest that TREM2 macrophages, predominantly found in L-lep lesions, differentiate to transitional macrophages, which are found in both RR and L-lep lesions, and further mature to ML4 macrophages, which are found predominantly in RR lesions. IL1B and IFNG are upstream regulators of the pseudotime trajectory in macrophages, acting as well on macrophage activation in granulomas to express genes known to contribute to antimicrobial responses. Focused on the spatial sequencing analysis, the surrounding zone of the granuloma is characterized by the presence of dendritic and Langerhans cells, along with T cell subtypes, which have the ability to deliver antimicrobial effector molecules into infected cells and can secrete cytokines that trigger a downstream antimicrobial response in macrophages. This analysis indicated that fibroblasts, keratinocytes and endothelial cells also express antimicrobial genes in RR granulomas. Moreover, two different fibroblast sub-populations were characterized, CXCL2^+^ fibroblasts and SFRP2^+^ fibroblasts. Localized at different sites of the periphery of the granuloma both fibroblast sub-populations contribute to the deposition of extracellular matrix proteins in the granuloma and to the release of antimicrobial proteins (Ma et al., 2021). These findings present a temporal and spatial model, not only of the immunological activation of multiple cell types, but also of the granuloma organization to effectively act in the control of pathogen activity.

Moreover, Carow et al. (2019) identified immune transcripts located within the tuberculosis granuloma using a highly sensitive multiplexed *in situ* imaging. The authors compared granulomas from lungs of mice at different time points after aerosol *M. tuberculosis* infection (3, 8, and 12 weeks). Although the similar transcription expression by innate immune cell (Tnf, Il6, Inos, Il12), an increase of transcripts related to T- or B cells (Cd3e, Cd19, Ccr6, Ifng) was observed at later time points. An over-representation of Cd19 mRNA was noted in the lymphoid cell area at 8 and 12 weeks. Cd19 and T-cell transcripts were co-expressed together with CCR6 mRNA in most lymphoid areas indicating close interactions between T cells and B cells. This data, together with the expression of Il12 mRNA in several lymphoid areas, highlights the importance of lymphocytes to orchestrate the immune response to *M. tuberculosis*. Moreover, in the epithelioid areas of the granuloma the main clusters included Cd68, Tnf, and Inos but also Cxcr3 and Ccr4 mRNA, expressed by T_*H*_1 and T_*H*_2 cells, respectively. The combined application of this technology with stained bacteria allowed the authors to take conclusions about the distance of different cellular types from the bacteria. Inos, Cd68, Cd11b, Tnf, and Socs3 mRNA were enriched at shorter distances to *M. tuberculosis*, suggesting that activated macrophages co-localize with this bacteria in the granuloma. On the other hand, transcripts such as Cc10, Spc, Il6, and Foxp3 were decreased at shorter distances. Other transcripts including Cd8b, Cd3e, Cxcr3, Ccr4, and Il12 showed similar frequencies at different distances from *M. tuberculosis*. Overall, this study highlights the potential of single-cell spatial analysis when combined with other techniques (Carow et al., 2019).

Although scRNA-seq has been extensively developed in recent years and answered different questions (as summarized in Table 2), little work has been done specifically on *M. ulcerans*, once no articles were found for this review. In contrast, the use of scRNA-seq in the field of *M. tuberculosis* is continuing to expand (Khan et al., 2020; Longo et al., 2021; Akter et al., 2022; Oelen et al., 2022). Thus, with the development of new techniques and the uptake of this method, it is imperative to take advantage of scRNA-seq to better understand the evolution of infection by identifying susceptible cell types, studying infection dynamics and immune inflammatory changes, discover biomarkers and, ultimately, unravel novel treatment strategies.

**Table 2:** Summary table containing the technique and major conclusions of the articles discussed in this section.

**Technique**	**Major conclusions**	**References**
Seq-Well	Genetic profile of macrophages exposed to *M. tuberculosis*	Gierahn et al., 2017
10x Chromium	Single-cell landscape of *M. tuberculosis*-infected macaques lungs during TB and latency	Esaulova et al., 2021
CITE-Seq	Differential abundance and function of T_*H*_17 subset between progressors and non-progressors donors	Nathan et al., 2021
10x Chromium	Identification of latent individuals with high risk to develop active TB disease based on monocytes immune cells	Bossel Ben-Moshe et al., 2019
10x Chromium	Identification of NK cell subset depleted, and monocytes and B cells increase during TB	Cai et al., 2020
CITE-Seq	Understanding of the roles that different host cell populations play during the course of an infection	Pisu et al., 2021
Seq-Well S3	Transcriptional landscape of inflammatory skin disease, leprosy	Hughes et al., 2020
10x Chromium	Primary suppressive landscape in the L-LEP patients	Mi et al., 2022
Seq-Well	Single-cell profiling of tuberculosis lung granulomas	Gideon et al., 2020
Seq-Well	Integration of scRNA-seq with spatial sequencing, to delineate the cellular and molecular structure of the organized granuloma in leprosy	Ma et al., 2021
*In situ* sequencing	Different immune landscapes of *M. tuberculosis* granulomas depending on the time after infection	Carow et al., 2019

## 5. Discussion

Mycobacteria are among the oldest and deadliest aetiologies of human infectious disease still accounting for large global morbidity and mortality. The current perspectives for mankind being able to control the negative burden of Mycobacterial diseases are not blooming. Innovation will be key for this defiant goal and will likely encompass a shift from the traditional chemotherapy to host-directed therapies and personalized medicine. For these to become a reality there is a strong need for deeper insights into the host immune status, intra-host pathogen evolution, and how it associates with different disease spectra in the course of Mycobacterial infection. Understanding the dynamic diversity within the populations of cells from the host and pathogen is fundamental to better understand protective immune responses and therapy resistance mechanisms. The high-resolution study of these processes is challenging but the technological developments in the field of single cell genomics bring new hope. The combination of incremental advances in recent years forms an idyllic vision for the future of Mycobacterial infection research than can be named “dual single-cell *in vivo* multiomics.” Dual RNA-seq emerged as powerful tool in which the simultaneous analysis of the host and pathogen transcriptomics added large value to dissect the interplay between the organisms. Most studies published to date in the field of Mycobacteria using dual RNA-seq focused on *M. tuberculosis* infection and are based on bulk analysis (Pisu et al., 2020). Exciting next steps will encompass taking these studies to the single cell level in primary cell cultures or tissue biopsies from animal models or the human host. Also brightly encouraging will be recording in each experiment the multiomics dimension by combining transcriptomics with other modalities such as epigenomics, metabolomics, or proteomics. As mentioned in this review scRNA-seq, along with ATAC-seq, spatial, and temporal transcriptomics were proven to be able to provide relevant insights into how cells behave and interact with adjacent cells through time in the context of the “natural” cellular niche and how they interplay in various cellular events and signaling cascades. These approaches performed in a dual(or multi)-organism approach can be of particular significance to better understand the dynamics of mycobacterial survival and growth within the host as these are often linked with cellular structures such as granulomas composed of heterogenous population of host and mycobacterial cells. Also potentially revolutionizing of the state-of-the-art will be to take into account the effects of the microbiome and syndemics by using these tools to investigate hosts affected by relevant global co-infections such as *M. tuberculosis* and HIV-1, SARS-CoV-2, or *Plasmodium falciparum*. Overall, these studies could decisively impact existing knowledge dissecting with unprecedented levels of detail the complex interplay between host and microbe cells influencing metabolism and other essential biological pathways and having a strong influence on disease outcome. Advances in single-cell genomics in the context of Mycobacterial infection also have the potential to impact future clinical practice helping clinicians to better prioritize and personalize treatment. In example, these methods will likely provide means for better detection/prevention of drug resistance and increase the accuracy of severe disease risk prediction. However, these approaches still encounter technical limitations that greatly challenge its expansion and scaling in research and development programmes. A major limitation in combining single cell with dual RNA-seq approaches for the simultaneous analysis of the host and mycobacterial transcriptome is the fact that the most validated scRNA-seq methods capture only polyadenylated RNA transcripts that are in low abundance in bacteria. The use of poly[T] priming in these methods for efficient RT discards all non-polyadenilated mycobacterial RNAs also excluding from the experiments host non-coding RNAs (such as microRNAs, long non-coding RNAs, circular RNAs, among others). Furthermore, the use of short-read NGS technologies for sequencing impairs the correct analysis of genomic regions that are highly repetitive or diverse. These regions often include molecular targets of high interest in the host-pathogen interaction including targets that are co-evolving to modulate immune responses or sites involved in evolution toward drug resistance. These could be surpassed by a more extensive validation of single cell genomics methods that do not rely on poly[T]/[A] priming and by the use of hybrid sequencing strategies combining short read with long read NGS technologies (Amarasinghe et al., 2020). Alongside these specific limitations the application of single cell genomics to the field of Mycobacterial infection is also affected by more general limitations such as: (i) the requirement for large volumes of sample, (ii) the need for more efficient cell isolation methods, (iii) the effect of sample preparation, processing, or storage on the obtained results, and (iv) the overall high costs. Moreover, albeit the advances and remarkable efforts in the development of bioinformatics for analysing single cell data the meaningful analysis of multiomics data, from multiple organisms within the same sample, incorporating temporal and spatial information is a major challenge requiring the development of improved computational methods (Chen et al., 2021; Cho et al., 2021; Fu et al., 2021). Confidently, in the long run, the advances in single cell genomics will generate findings translatable into novel therapeutic targets, biomarkers of prognosis, ways to track and prevent transmission ultimately bringing us closer to eradicate the devastating diseases caused by Mycobacteria.

## Author contributions

NO and IG designed the study. MF prepared the figures. IG, MF, AF, and NO wrote the manuscript. All authors have read and agreed to the published version of the manuscript.

## Funding

This work has been funded by Portuguese National funds, through the Foundation for Science and Technology (FCT)—project UIDB/50026/2020 and UIDP/50026/2020.

## Conflict of interest

The authors declare that the research was conducted in the absence of any commercial or financial relationships that could be construed as a potential conflict of interest.

## Publisher's note

All claims expressed in this article are solely those of the authors and do not necessarily represent those of their affiliated organizations, or those of the publisher, the editors and the reviewers. Any product that may be evaluated in this article, or claim that may be made by its manufacturer, is not guaranteed or endorsed by the publisher.
